# Mechanism of SC targeting RhoA regulation and its potential value in gastric cancer therapy

**DOI:** 10.1016/j.bbrep.2025.102158

**Published:** 2025-07-24

**Authors:** Haixiu Ma, Ping Jiang, Ronghua Ma, Jing Zhao, Qi Wang, Yonghua Xing, Chengzhu Cao, Zhanhai Su

**Affiliations:** a Research Center for High Altitude Medicine, Key Laboratory for High Altitude Medicine, Ministry of Education, Qinghai University, 16# Kunlun Road, Xining, 810016, Qinghai, China; b Department of Basic Medicine Science, Qinghai University Medical College, Xining, 810001,Qinghai, China

**Keywords:** Dual action, Drug discovery, RhoA inhibitor, EMT, Natural product

## Abstract

RhoA drives the malignant progression of gastric cancer through cytoskeletal remodeling and the regulation of epithelial-mesenchymal transition (EMT). Here, we identified a novel small-molecule inhibitor, (E)-1,9-bis(3,4-dihydroxyphenyl)non-3-en-5-one (SC), targeting RhoA through molecular docking and surface plasmon resonance (SPR) validation. SPR kinetics revealed high-affinity binding (KD = 1.588 μM) with rapid association (ka = 2.769 × 10^3^ 1/Ms) and slow dissociation (kd = 4.398 × 10^−3^ 1/s), achieving stable SC-RhoA complex formation. In vitro, SC suppressed RhoA expression, in turn upregulating E-cadherin, downregulating N-cadherin and Vimentin, and inhibiting cell migration (*p* < 0.001). Scanning electron microscopy confirmed pseudopodia retraction and cytoskeletal collapse. Remarkably, oral administration of SC (50 mg/kg/day) attenuated tumor growth in a xenograft model. These results present SC as a potential dual-action RhoA inhibitor that concurrently disrupts GTPase activity and protein stability, offering a promising therapeutic strategy against gastric cancer.

## Highlights

• Identification of SC as the monomeric compound with specific anti-gastric cancer activity.
• SC triggers significant cell death in gastric cancer cells.
• Dual-targeting capability by coupling enzymatic inhibition with protein destabilization.
• SC might as a RhoA-directed therapeutic for gastric cancer.

## 1 Introduction

The global burden of cancer persists as a critical public health challenge, with over 19 million new cases reported in 2022. Gastric cancer (GC) represents a particularly aggressive malignancy, ranking as the fourth leading cause of cancer-related mortality worldwide [1]. While surgical resection remains the cornerstone of localized GC treatment, approximately 60 % of patients present with advanced or metastatic disease at diagnosis, necessitating systemic therapeutic interventions [2]. This clinical urgency underscores the imperative for novel molecularly targeted therapies to address the limitations of current treatment paradigms.

Central to cancer progression, Rho GTPases orchestrate cytoskeletal dynamics [3] and metastatic dissemination through multifaceted mechanisms [4]. Among these, RhoA serves as a critical molecular hub, regulating actomyosin contractility via ROCK-mediated myosin light chain phosphorylation and mDia-dependent actin polymerization [5]. Mechanistically, RhoA integrates mechanical cues via the integrin-ROCK/YAP axis to drive EMT, while its functional loss paradoxically activates Wnt/β-catenin signaling to destabilize intercellular junctions [6]. This mechanical-transcriptional coupling enables tumor cells to dynamically adapt to heterogeneous microenvironments during metastatic progression [7]. Various RhoA inhibitors have been reported, with Rhosin, which specifically binds to RhoA, being the first RhoA subfamily-specific inhibitor developed to target GEF activation, and has been used in different cancer cells [[8], [9], [10]]. C3 exotoxin from Clostridium botulinum, Grincamycin B, and Riboprine (N6-isoprenyladenosine) inhibit RhoA activity, in turn suppressing tumor growth and invasion [3,[11], [12], [13]]. CCG-100,602 and CCG-203,971 are new RhoA inhibitors that have lower toxicity, higher selectivity, and greater efficacy than CCG-1423[14]. In addition, PTX-100 is a GGT-1 inhibitor that inhibits the prenylation process of RhoA and is currently undergoing a Phase 1 clinical trial for patients with advanced diseases (NCT03900442) [15].

Despite its therapeutic potential, RhoA presents unique pharmacological challenges. Structural analyses reveal a globular conformation lacking druggable pockets beyond its conserved nucleotide-binding domains, with picomolar affinity for GDP/GTP that approaches the thermodynamic limits of competitive inhibition. Clinical translation of RhoA inhibitors has been further hampered by poor bioavailability and rapid proteolytic degradation, exemplified by the pharmacokinetic limitations of C3 transferase [16]. These constraints highlight the critical need for innovative targeting strategies that combine enzymatic modulation with enhanced molecular stability.

Our current investigation identified SC as an orally bioavailable potential RhoA modulator isolated from *Saxifraga tangutica Engl.* SC demonstrated dual functionality as both a RhoA-binding agent and proteolysis-targeting chimera. This discovery builds upon traditional applications of *Saxifraga tangutica Engl.* in Tibetan medicine for hepatic disorders [17] and research of its extracts on GC cells [18], while addressing a critical gap in understanding its bioactive constituents. Unlike previous studies that used crude extracts [19], our work suggests that SC may have potential specific anti-GC activity, providing further data to support its therapeutic potential.

## 2 Materials and methods

### 2.1 Isolation and purification of the compounds from Saxifraga tangutica Engl

*Saxifraga tangutica Engl* used in this study was collected in the Tibetan Autonomous Prefecture of Guoluo, Qinghai Province. The corresponding specimen is currently preserved in the Qinghai-Tibet Plateau Biodiversity Museum (Specimen No. 0325734). Specimens were air-dried under cool conditions, and a 33 kg sample was selected for extraction, using 100 % methanol, by heating and refluxing three times at 60 °C. The resulting extracts were filtered and concentrated by rotary evaporation under reduced pressure to yield approximately 1897.9 g of herbal extract. This extract was then subjected to 7-X10 preparative chromatography column (20 × 250 mm, 5 μm), silica gel column chromatography, polyamide, and gel, yielding compounds 1 (yellow oily substance), 2 (yellow oily substance), 3 (off-white solid), 4 (off-white solid), 5 (off-white solid), and 6 (off-white solid). The structural elucidation of these compounds was conducted through mass spectrometry (Manufacturer: Waters Corporation, USA; Model: Quattro Premier XE), ^1^H nuclear magnetic resonance (NMR), and ^13^C NMR techniques (Swiss Bruker Company Model 400 M: BRUKER AVANCE III HD 400 MHz). Importantly, the purity of the extracted compounds was > 98 %.

### 2.2 Cell culture

The cells were cultured in Dulbecco's Modified Eagle Medium (DMEM) (ProCell; Wuhan, China) supplemented with 10 % fetal bovine serum (FBS) (ProCell), l-glutamine at a concentration of 2 mM, and 1 % penicillin/streptomycin (ProCell). The culture was incubated at 37 °C in a 5 % CO_2_ atmosphere. The HGC-27 and AGS cell lines were procured from ProCell.

### 2.3 Cell survival assays

The CCK-8 assay involved plating cells (0.2–0.3 × 10^4^ cells/well) in 96-well plates, which were then incubated in a CO_2_ atmosphere at 37 °C. The cells were exposed to varying concentrations of SC for 24 h to determine the IC_50_. Subsequently, the cells were incubated with CCK-8 (Cell Counting Kit-8) in the absence of light at 37 °C for 2–4 h, and the optical density (OD) was measured at 460 nm using a SpectraMax Paradigm Multi-Mode Detection Platform.

### 2.4 Western blotting

Cells were plated in 60-mm plates at a density of 3–5 × 10^5^ cells and incubated in a CO_2_ atmosphere at 37 °C. Following SC treatment, the cells were lysed using RIPA lysis buffer (Solarbio) supplemented with a protease inhibitor (Thermo Scientific). The lysates were then boiled at 100 °C for 10 min, and the protein contents were quantified using the Pierce BCA Protein Assay Kit (Thermo Scientific). Equal amounts of proteins (10 μg per lane) were sonicated and subjected to analysis using SDS-PAGE (10 % and 15 % for determination of β-actin (proteintech, 66009-1-1g, 1:20000), Vimentin (proteintech, 10366-1-AP,1:2000), N-cadherin (proteintech, 66219-1-lg,1:5000), E-cadherin (proteintech, 20874-1-AP,1:5000), and RhoA (proteintech, 10749-1-AP,1:500). The proteins were subsequently transferred onto polyvinylidene fluoride membranes (Millipore, Bedford, MA, USA). Immunoblotting was performed using the aforementioned antibodies, followed by incubation with a secondary antibody conjugated to horseradish peroxidase for 2 h at room temperature. Following three washes with tris buffered saline with tween 20, enhanced chemiluminescence Pierce ECL Western Blotting Substrate (Abkine, Wuhan, China) was used to detect the signals, which were then captured using a Kodak Image Station 2000R.

### 2.5 Transwell assay

Cells were plated onto the upper chambers of transwell plates (8-μm pore-size filter membranes; Corning Inc., New York, NY, USA) in serum-free medium, while the medium containing 10 % FBS was added to the lower chambers. Transwell plates were then placed in a CO_2_ incubator at 37 °C. Cells that migrated to the bottom of the membranes were fixed with 4 % paraformaldehyde and stained with 1 % crystal violet (Macklin, Shanghai, China). The cells were imaged under a microscope, and the number of migrated cells was determined by counting five random fields.

### 2.6 Immunofluorescence staining

Cells were plated in 6-well plates at a density of 3–5 × 10^4^ cells per well and incubated in a CO_2_ atmosphere at 37 °C. Following treatment, the cells were fixed in 4 % paraformaldehyde (PFA) from Servicebio (Wuhan, China) for 15 min and subsequently washed three times with PBS from ProCell. We applied 500 μL of 0.5 % Triton-X 100 (Biosharp, Beijing, China) for 15 min to permeabilize the cells, which were then blocked with 10 % FBS-PBS for 2 h at room temperature. The cells were incubated overnight at 4 °C with primary antibodies against F-actin (dilution of 1/100, ab16895, Abcam, Cambridge, UK), E−cadherin (dilution of 1/200, proteintech, 20874-1-AP), and N-cadherin (dilution of 1/200, proteintech, 66219-1-lg). The cells were washed with PBS for 3 × 5 min. Subsequently, the cells were incubated with the secondary antibodies necessary for indirect immunofluorescence at room temperature for 2 h. Nuclei were stained with 30–50 μL of 4′,6-diamidino-2-phenylindole (DAPI) (Solarbio, Beijing, China) for 5 min. The resulting images were examined using a Nikon A1R + laser confocal microscope (Nikon, Japan).

### 2.7 Molecular docking

Molecular docking analysis was performed using SwissDock software (http://www.swissdock.ch/). The crystal structures of RhoA (PDB ID: 1KMQ) were sourced from Protein Data Bank (PDB). The molecular docking results included predicted docking visualizations. The binding energy (kcal/mol) was utilized to evaluate the binding affinity of the cluster, with a lower binding energy value indicating a stronger affinity. Additionally, the molecular docking of SC to proteins was performed using MOE (Version 2014.09, Chemical Computing Group Inc., Cambridge, UK) to predict the binding sites of SC to the proteins.

### 2.8 Surface plasmon resonance (SPR) assay

The binding affinity between indicated SC and RhoA proteins was detected by Biacore T200 Cytiva. RhoA proteins (30–50 μg/mL) were dissolved in PBS and immobilized onto a Series S Sensor Chip CM5 Cytiva (Sweden, BR100012 LOT 10344853). The SC diluted to a running buffer composed of 5 % DMSO in PBS. Then, different concentrations (25-12.5-6.25-3.125-1.5625–0.78125 μM) of SC were injected into the instrument and flown over the CM5 chip to produce response signals. The binding affinity, represented by the KD value, was evaluated using Biacore T200 Evauation Software 3.0.

### 2.9 Scanning electron microscope (SEM)

Following treatment with SC, the cells were subjected to fixation with 3 % glutaraldehyde at 4 °C for an overnight duration. Subsequently, dehydration was performed using increasing concentrations of ethanol, followed by thorough drying using a vacuum system. The uniform deposition of a thin layer of gold onto the cell surface was achieved using a sputter coater. The prepared samples were subsequently introduced into an SEM instrument (JEM-2100F), where electron beams were used to scan the cell surfaces and capture the resulting electron images.

### 2.10 In vivo experiments

BALB/c nude mice (4 weeks, 20 male and 20 female) were purchased from Beijing Vital River Laboratory Animal Technology Co., Ltd. The animals were housed in a pathogen-free facility at the Animal Center of Qinghai University. The housing room was set to a 12-h/12-h light/dark cycle, and all animals had access to a chow diet and water. After tumor transplantation, the tumors were observed after 7 days, and mice were further divided into subgroups (control (200 μL of 0.25 % DMSO, gastric lavage), low-dose group of SC (12.5 mg/kg/day, gastric lavage), middle-dose group of SC (25 mg/kg/day, gastric lavage), and high-dose group of SC (50 mg/kg/day, gastric lavage). After 21 days of treatment, the mice were euthanized through spinal dislocation. The maximum tumor size of the experimental endpoint mouse model was <20 mm in all dimensions.

### 2.11 Statistical methods

Data were statistically analyzed using SPSS 28.0. Two-group comparisons were conducted using t-tests and more than three groups were analyzed using one-way analysis of variance. Non-normally distributed data were evaluated for group differences using rank-sum tests, with a significance level of *α* = 0.05 (two-tailed).

### 2.12 Ethics approval

The animal care and experimental protocols were approved by the Research Ethics Committee of Qinghai University, with the ethical review number: PJ292404-13, and were conducted in strict accordance with the ethical guidelines of the Ethics and Safety Committee of Qinghai University.

## 3 Results

### 3.1 SC directly interacts with RhoA protein and effectively inhibits its activity in vitro

The MS, ^1^H, and ^13^C NMR spectral data of compound 5 is provided in the supplementary materials (Fig. S1A–B). MS data shows formation of a main ion peak at *m/z* 357 [M+H]+, and an alkali metal adduct ion with sodium (Na+) at *m/z* 379 [M+Na]+. Additionally, a main ion peak was observed at *m/z* 355 [M − H]-, suggesting a molecular weight of 356 for the compound. Based on these findings, the molecular formula of the compound is inferred to be C_21_H_24_O_5_. ^1^H NMR (400 M Hz, DMSO‑*d*_6_) shows δ:8.66 (brs,4H,-OH), 6.41–6.65 (m,6H,H–Ar), 6.07 (d, *J* = 15.9 Hz,1H,H-6), 6.82 (m,1H,H-7), 2.40–2.57 (m,8H), and 1.47 (brs,4H,H-2/3). ^13^C NMR (100 M Hz,DMSO‑*d*_6_) shows δ:34.8(C-1), 31.2(C-2), 23.9(C-3), 39.5(C-4), 200.4(C-5), 130.8(C-6), 147.2(C-7), 33.5(C-8), 34.3(C-9), 133.3, 132.2(C-1’/1″), 116.2, 116.1(C-2’/2″), 145.5, 145.4 (C-3’/3″), 143.8, 143.6(C-4’/4″), 115.9, 115.9(C-5’/5″), 119.4, and 119.3(C-6’/6″). These data are essentially consistent with those reported for compound (7) in the literature [20], and thus the compound is identified as Saxitansin C, with its chemical structure depicted in Fig. 1. Molecular docking was deployed to assess the binding potential of SC with RhoA. The results reveal that SC interacts with the Switch I region and the α5-helix of RhoA, demonstrating dual binding to functionally distinct structural motifs. Within the Switch I region (yellow-green area, Fig. 2A), TYR-34 engages in π-π stacking interactions (3.9 Å, purple dashed line) and a π-σ interaction (3.6 Å) with PHE-30, forming a hydrophobic core (purple highlight, Fig. 2B). Concurrently, THR-37 establishes a robust hydrogen bond with the ligand (2.8 Å, green dashed line), a configuration that directly competes with the binding site of the γ-phosphate group of GTP (red highlight, Fig. 2A). This competitive interaction suggests steric and electronic hindrance to GTP binding in the active site. In the α5 helix (highlighted in blue in Fig. 2A), disruption of the salt bridge between LYS-162 and ASP-120 (distance: 3.4 Å, blue dashed line) induces conformational relaxation of the α5 helix. Molecular dynamics simulations further reveal that ALA-161 occupies the hydrophobic pocket via van der Waals interactions (3.2–3.6 Å, light green surface in Fig. 2B), thereby stabilizing the conformational integrity of the Switch II region. The SPR assay showed that in the binding phase (0–150 s), a concentration-dependent increase in response units occurred, fitting a 1:1 Langmuir binding model (R^2^ > 0.99) with no evidence of aggregation or multivalent interactions, followed by a dissociation phase (150–250 s) characterized by minimal signal decay and a low dissociation rate constant (kd = 4.398 × 10^−3^), consistent with high binding stability (Fig. 2C).

**Fig. 1:**
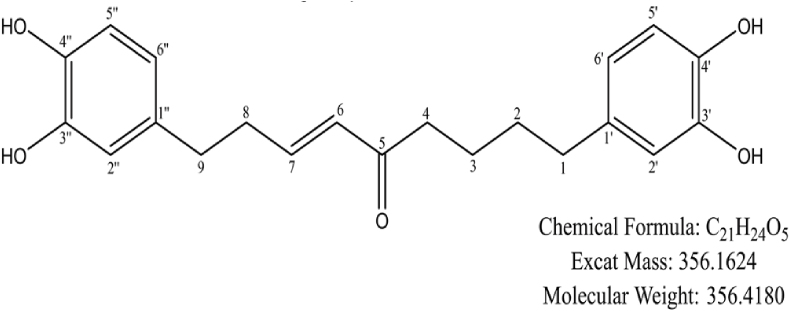
Chemical structure of SC.

**Fig. 2:**
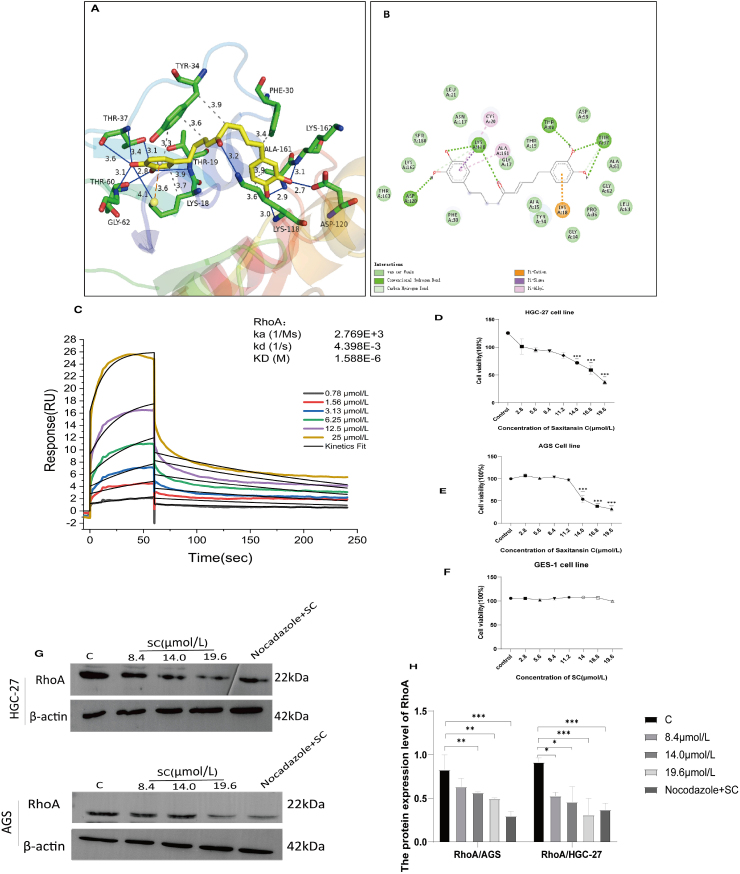
Molecular interaction mechanism between SC and RhoA. (A–B) Analysis of molecular docking results. (C) An SPR assay was used to predict the binding affinity of SC to RhoA. (D–F) HGC-27, AGS, and GES-1 cells were treated with various concentrations of SC for 24 h and cell viability was determined using the CCK-8 assay (n = 3). (G) HGC-27 and AGS cells were treated with varying concentrations of SC with or without nocodazole (50 ng/mL) for 24 h. (H) RhoA protein levels were assessed using western blotting (n = 3). ∗*P* < 0.05, ∗∗*P* < 0.01 vs. the Control group.

To further elucidate the potential role of SC in the inhibition of RhoA, we conducted a CCK-8 assay and western blotting. The CCK-8 assay revealed significant anticancer activities solely for SC, with IC_50_ values of 14.03 and 14.1 μmol/L in HGC-27 and AGS cells, respectively (Fig. 2D and E).In addition, SC was not significantly cytotoxic to normal cells such as GES-1 (Fig. 2F). Western blotting confirmed that SC effectively inhibited RhoA expression level in HGC-27 and AGS cells (Fig. 2G and H). Studies have shown that the activity of RhoA is significantly increased in cells treated with nocodazole [21]. In this study, the suppressive effect of SC on RhoA expression in these cells was partially reversed by the activation of RhoA following nocodazole treatment.

### 3.2 SC disrupts cytoskeletal dynamics of HGC-27 and AGS cells

Staining revealed that in control cells, dense filamentous stress fibers at cellular edges and cytoplasmic longitudinal fiber bundles (Fig. 3A, F-actin) were representative of an active cytoskeletal architecture, whereas SC treatment (19.6 μmol/L) induced actin network disassembly manifested by fragmented stress fibers and diffuse granular fluorescence (Fig. 3A, F-actin of 19.6 μmol/L). Control cells exhibited cytoplasmic-dominant E-cadherin localization with weak membrane discontinuity (Fig. 3A, E-cadherin), characteristic of mesenchymal phenotypes, while SC-treated cells displayed reinforced continuous membrane junctions with signals, indicative of epithelial phenotype restoration. We observed notable morphological alterations in HGC-27 and AGS cells through scanning electron microscopy (Fig. 3B). Scanning electron microscopy revealed that low-concentration SC (8.4 μmol/L) preserved filopodia on AGS/HGC-27 cell surfaces (2000 × ), correlating with partial F-actin integrity in immunofluorescence controls; at 19.6 μmol/L, AGS cells exhibited smooth membrane edges (2000 × ) indicative of stress fiber disassembly, while HGC-27 cells displayed narrowed intercellular gaps (500 × ), consistent with E-cadherin relocalization and restored adhesion junctions.

**Fig. 3:**
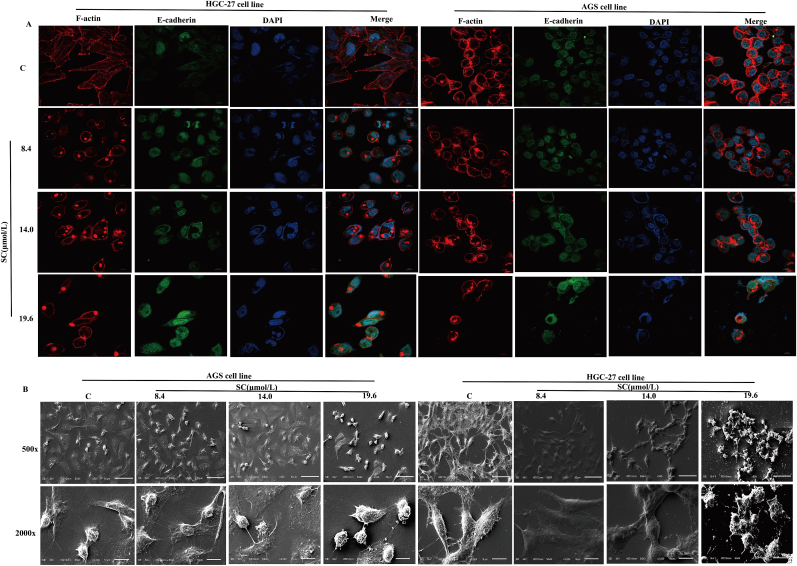
SC disrupts cytoskeletal dynamics of HGC-27 and **AGS cells.** (A) Distribution and localization of F-actin and E-cadherin in gastric cancer cells after SC treatment; Blue: DAPI, Red: F-actin, Green: E-cadherin. (B) HGC-27 and AGS cells were treated with 8.4, 14.0, and 19.6 μmol/L SC for 24 h. After treatment, the cells were fixed with a glutaraldehyde fixing solution, and representative images of cell morphology and pseudopods were captured using a SEM (n = 3).

### 3.3 SC inhibits migration, invasion, and EMT potential of gastric cancer cells in vitro

**Fig. 4:**
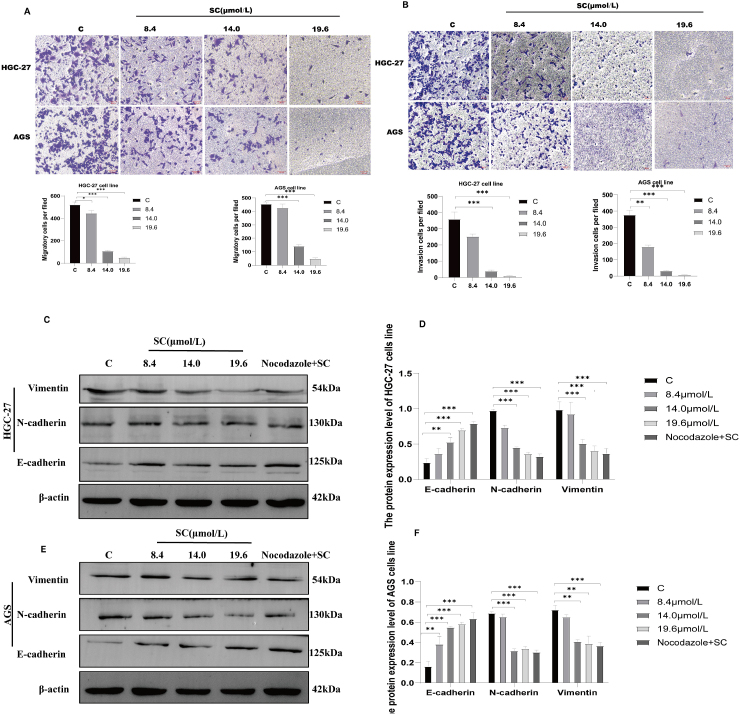
Effect of SC on migration and invasion ability. (A–B): SC inhibits the migration and invasion of HGC-27 and AGS cells. Cells were treated with 8.4, 14.0, and 19.6 μmol/L SC for 24 h in the upper chambers with (invasion, 3 × 10^5^ cells were plated onto the upper chambers) or without (migration, 1 × 10^5^ cells were plated onto the upper chambers) Matrigel, while the lower chambers were filled with complete DMEM. The images of migrated and invaded cells were captured after fixation with 4 % PFA and staining with crystal violet. Scale bar = 20 μm. (C, E): HGC-27 and AGS cells treated with or without nocodazole for 24 h; the protein levels of E-cadherin, N-cadherin, and Vimentin were determined by western blotting (n = 3). (D, F) Quantitative analysis of individual proteins. ∗∗*P* < 0.01, ∗∗∗*P <* 0.001 vs C (control group).

Metastasis is the leading cause of mortality among a significant number of patients with cancer, with the migratory and invasive properties of cancer cells forming the cornerstone of metastatic progression [22]. To assess the impact of SC on the migratory and invasive abilities of cancer cells, transwell migration and invasion assays were performed. Treatment of the cells with SC at concentrations of 8.4, 14.0, and 19.6 μmol/L resulted in a significant decrease in cancer cell migration (Fig. 4A). Furthermore, the invasive capability of these cells was substantially impaired (Fig. 4B). Western blotting analysis demonstrated that SC treatment induces dose-dependent alterations in EMT markers: E-cadherin expression was significantly upregulated (Fig. 4C–F, *p* < 0.001), while N-cadherin and Vimentin levels exhibited marked downregulation (Fig. 4C–F, *p* < 0.001), collectively suggesting SC-mediated EMT reversal, loss of mesenchymal phenotype, and consequent suppression of cell migratory capacity.

### 3.4 SC inhibits gastric cancer growth in vivo

**Fig. 5:**
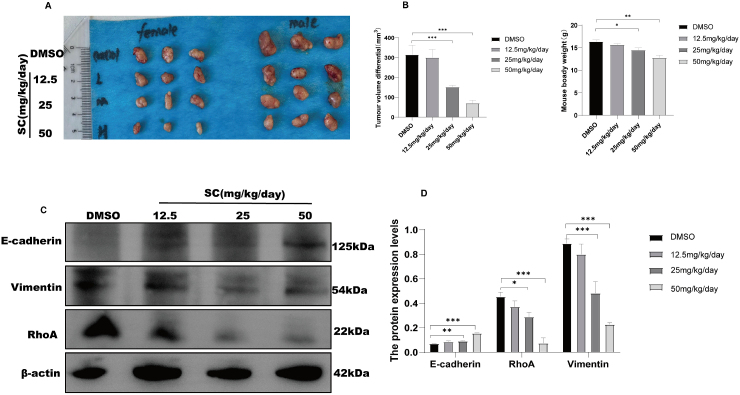
SC inhibits gastric cancer growth in vivo. (A) Schematic representation of tumor size in mice treated with different concentrations of SC (n = 6). (B) Change in tumor volume and body weight. (C) Western blotting analysis of E-cadherin, Vimentin, and RhoA expression in mice. (D) Statistical analysis of C. ∗*P* < 0.05, ∗∗*P* < 0.01 vs. the Control group.

We further explored whether SC influences the growth capabilities of gastric cancer cells by targeting the cytoskeleton in vivo (Fig. 5). To this end, a gastric cancer mouse model was successfully established by subcutaneously injecting MKN-45 cells (7 × 10^6^ cell/mice) into each animal for 1 week. Subsequently, mice in different groups were administered SC via gastric lavage at doses of 12.5, 25, or 50 mg/kg/day for 2 weeks. The tumor size in the SC treatment groups progressively decreased with increasing SC concentration, generally exhibiting a dose-dependent inhibition of tumor proliferation (Fig. 5A and B). SC significantly reduced RhoA and vimentin production in the treatment groups, whereas E-cadherin expression increased at higher SC doses. This upregulation of E-cadherin was likely due to the inhibition of RhoA expression, which in turn activated E-cadherin expression (Fig. 5C and D), indicating a suppression effect of SC on RhoA and Vimentin. No significant lesions were observed in other tissues, consistent with our in vitro findings; these data are shown in supplementary materials(Fig.S2) (Fig. S2A–B). These results further elucidate that SC likely exerts its antitumor activity by targeting RhoA to modulate cytoskeletal dynamics and reverse EMT, thereby suppressing tumor cell proliferation.

## 4 Discussion

*Saxifraga tangutica Engl*, though previously understudied for its anti-gastric cancer properties, has demonstrated compelling results in recent research. SC, a compound derived from *Saxifraga tangutica Engl*, has shown significant inhibitory effects on GC cells. Research reported that quercetin, isolated from *Saxifraga tangutica Engl*, significantly inhibits the growth of the human GC cell line (BGC-823) in a dose- and time-dependent manner [18]. Our study identified that SC could be a novel RhoA inhibitor with potential to suppress GC metastasis through blocking two critical processes: cytoskeletal reorganization and EMT plasticity. Using molecular docking, functional experiments, and high-resolution imaging, we showed that SC disrupts RhoA-driven metastatic pathways, providing a mechanistic foundation for its therapeutic potential in advanced cancers.

Herein, SC exhibited high-affinity binding to RhoA (KD = 1.588 μM), targeting both the Switch I domain (mimicking the inactive GDP-bound state) and the α5 helix. This dual engagement defines a two-pronged inhibitory mechanism: Switch I occupancy sterically blocks GTPase effector recruitment [23], while α5 helix interaction induces structural destabilization, priming RhoA for ubiquitin-proteasome degradation [24]. Dose-dependent RhoA depletion (in vitro/in vivo) confirmed this degradation-competent mode, contrasting with conventional GTP-competitive inhibitors that spare total RhoA levels. We speculate that the decrease in RhoA protein expression could be due to ubiquitin-proteasome degradation. Our SEM images provided striking spatial resolution confirming the effects of SC; this was aligned with observed F-actin fragmentation in immunofluorescence. These changes mirror ROCK/LIMK/cofilin pathway inhibition-reduced *p*-MLC and activated cofilin severing actin filaments [25].

Notably, while the microtubule inhibitor nocodazole reduced 2D migration, its inability to inhibit 3D invasion or reverse EMT highlights the unique advantage of SC in targeting actin-driven metastatic programs. The dual modulation of cytoskeletal dynamics and transcriptional reprogramming is exemplified by its EMT-suppressive effects. As demonstrated in Fig. 4, SC treatment restored E-cadherin expression while suppressing N-cadherin and vimentin, likely mediated through RhoA inhibition-induced disruption of the TAZ-Snail transcriptional axis [26]. This mechanism relieves repression of the CDH1 promoter (encoding E-cadherin), suggesting that RhoA depletion promotes epithelial restitution by rebalancing EMT-regulatory networks. The concordance between EMT marker reversal, morphological shifts, and functional suppression establishes SC as a true EMT inhibitor which may be a property absent in cytoskeletal drugs like nocodazole. The antitumor efficacy of orally administered SC (50 mg/kg/day) was evidenced by significant suppression of tumor proliferation and reduced xenograft volume without detectable toxicity, supporting its preclinical relevance in GC therapeutic development.

While our mechanistic data nominate RhoA inhibition as a translationally actionable node in GC management, two critical knowledge gaps remain unresolved. First, RhoAG17V mutations of gastric carcinomas [27] may confer structural resistance to canonical inhibitors like SC by stabilizing GTP-bound RhoA, necessitating mutant-allele-specific therapeutic development. Second, the anti-tumor efficacy of SC requires validation in patient-derived primary GC cells and 3D organoid models to assess clinical translatability across heterogeneous tumor microenvironments. Future efforts will prioritize engineering SC-loaded nanoparticle formulations (e.g., PEG-PLGA carriers) [28] to enhance tumor targeting efficiency and mitigate off-tumor RhoA modulation in normal tissues, and further pharmacokinetic optimization and toxicity profiling are warranted to advance this strategy toward clinical trials.

## 5 Conclusion

Our mechanistic findings position SC as an innovative RhoA inhibitor that concurrently dismantles the cytoskeletal machinery of invasion and epigenetically locks tumors in an epithelial state. Its ability to bridge structural biology with transcriptional reprogramming offers foundational data support for future research.

## CRediT authorship contribution statement

**Haixiu Ma:** Writing – review & editing, Writing – original draft, Conceptualization. **Ping Jiang:** Writing – review & editing. **Ronghua Ma:** Methodology, Conceptualization. **Jing Zhao:** Software. **Qi Wang:** Formal analysis. **Yonghua Xing:** Supervision, Data curation. **Chengzhu Cao:** Visualization. **Zhanhai Su:** Writing – review & editing, Project administration, Funding acquisition.

## Declaration of generative AI and AI-assisted technologies in the writing process

During the preparation of this work, the author(s) used [Kimi.ai] to [improve language and readability]. After using this tool, the author(s) reviewed and edited the content as needed and take(s) full responsibility for the content of the publication.

## Funding sources

This research was supported by 10.13039/501100012579Natural Science Foundation of Qinghai Province of China (2023-ZJ-932 M), 10.13039/501100001809National Natural Science Foundation of China (32360242), and 10.13039/501100010230Natural Science Foundation of Qinghai University Medical College of China (2022-kyy-4).

## Declaration of competing interest

The authors declare the following financial interests/personal relationships which may be considered as potential competing interests: Zhanhai Su reports was provided by atural Science Foundation of Qinghai Province of China. Zhanhai Su reports a relationship with 10.13039/501100010230Qinghai University that includes: funding grants. If there are other authors, they declare that they have no known competing financial interests or personal relationships that could have appeared to influence the work reported in this paper.

## Figures and Tables

**figs1:**
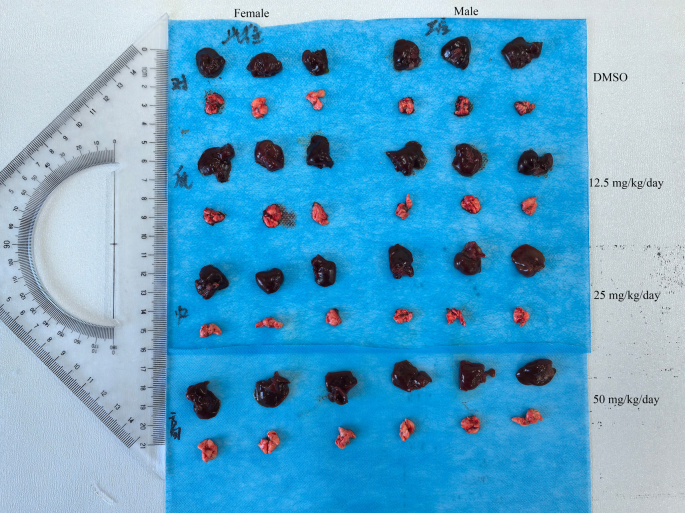


**figs2:**
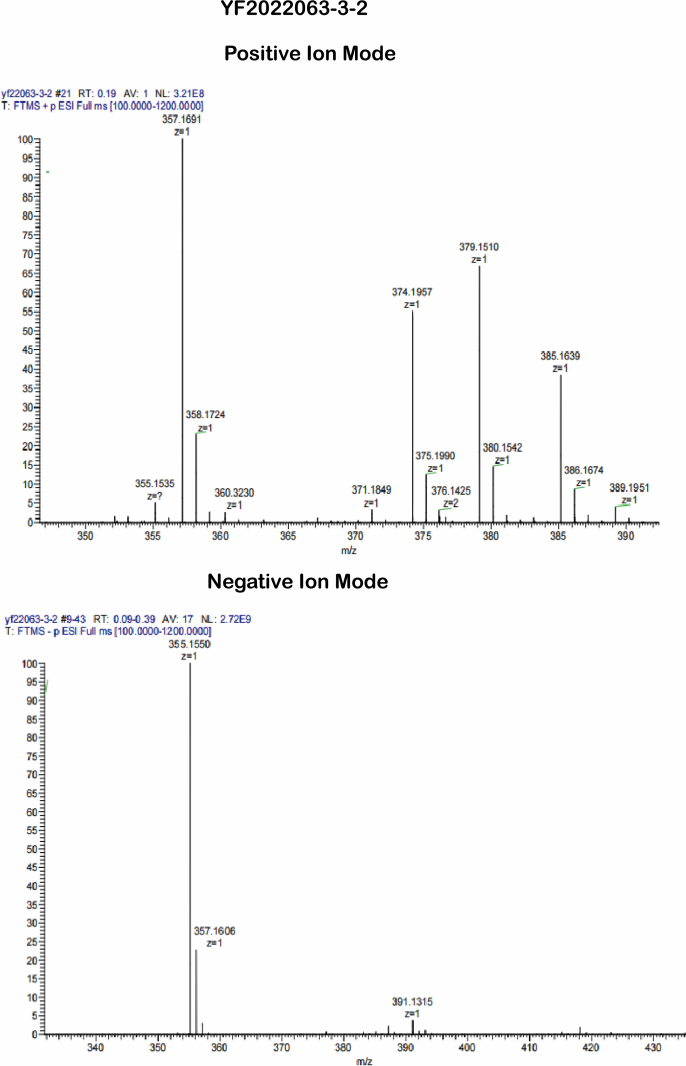


**figs3:**
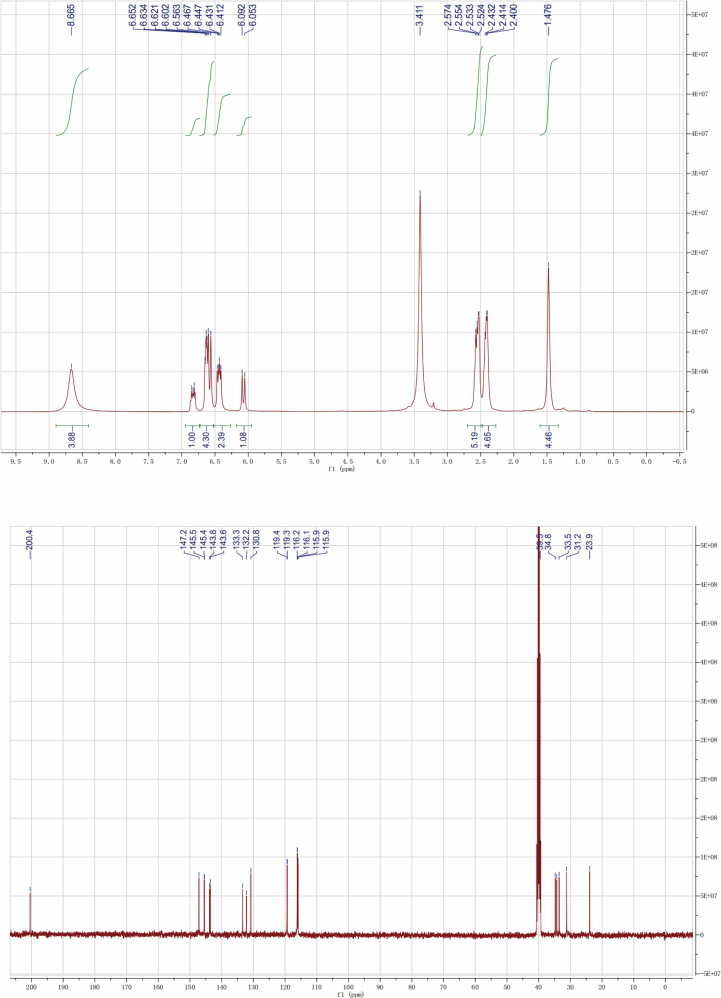


## Data Availability

All data are presented in the article.

## References

[1] Mao J.J., Pillai G.G., Andrade C.J. (2022). Integrative oncology: addressing the global challenges of cancer prevention and treatment. CA Cancer J. Clin..

[2] López M.J., Carbajal J., Alfaro A.L. (2023). Characteristics of gastric cancer around the world. Crit. Rev. Oncol. Hematol..

[3] Sun Z., Zhang H., Zhang Y. (2020). Covalent inhibitors allosterically block the activation of rho family proteins and suppress cancer cell invasion. Adv. Sci. (Weinh.).

[4] Sadok A., Marshall C.J. (2014). Rho GTPases: masters of cell migration. Small GTPases.

[5] Bustelo X.R. (2018). RHO GTPases in cancer: known facts, open questions, and therapeutic challenges. Biochem. Soc. Trans..

[6] Liu Z., Li S., Qian X., Li L., Zhang H., Liu Z. (2021). RhoA/ROCK-YAP/TAZ axis regulates the fibrotic activity in dexamethasone-treated human trabecular meshwork cells. Front. Mol. Biosci..

[7] Cambria E., Coughlin M.F., Floryan M.A., Offeddu G.S., Shelton S.E., Kamm R.D. (2024). Linking cell mechanical memory and cancer metastasis. Nat. Rev. Cancer.

[8] Shang X., Marchioni F., Sipes N. (2012). Rational design of small molecule inhibitors targeting RhoA subfamily rho GTPases. Chem Biol.

[9] Mu G., Ding Q., Li H. (2018). Gastrin stimulates pancreatic cancer cell directional migration by activating the Gα12/13–RhoA–ROCK signaling pathway. Exp. Mol. Med..

[10] Tsubaki M., Genno S., Takeda T. (2021). Rhosin suppressed tumor cell metastasis through inhibition of Rho/YAP pathway and expression of RHAMM and CXCR4 in melanoma and breast cancer cells. Biomedicines.

[11] Liang J, Huang J, Yang J, et al. Synthesis and in vitro evaluation of benzo[b]thiophene-3-carboxylic acid 1,1-dioxide derivatives as anticancer agents targeting the RhoA/ROCK pathway. J. Enzym. Inhib. Med. Chem. 39(1):2390911. doi:10.1080/14756366.2024.2390911. 10.1080/14756366.2024.2390911 PMC11391881 39258708

[12] Magalhaes Y.T., Cardella G.D., Forti F.L. (2020). Exoenzyme C3 transferase lowers actin cytoskeleton dynamics, genomic stability and survival of malignant melanoma cells under UV-light stress. J. Photochem. Photobiol. B Biol..

[13] Yao Y., Sun S., Cao M. (2020). Grincamycin B functions as a potent inhibitor for glioblastoma stem cell via targeting RHOA and PI3K/AKT. ACS Chem. Neurosci..

[14] Johnson L.A., Rodansky E.S., Haak A.J., Larsen S.D., Neubig R.R., Higgins P.D.R. (2014). Novel Rho/MRTF/SRF inhibitors block matrix-stiffness and TGF-β–Induced fibrogenesis in human colonic myofibroblasts. Inflamm. Bowel Dis..

[15] Tnimov Z., Abankwa D., Alexandrov K. (2014). RhoGDI facilitates geranylgeranyltransferase-I-mediated RhoA prenylation. BBRC (Biochem. Biophys. Res. Commun.).

[16] Santiago-Lopez A.J., Gutekunst C.A., Gross R.E., Rivero F. (2018). *Rho Gtpases*. Vol 1821. Methods in Molecular Biology.

[17] Li Y., Jia L., Zhang F., Wang Z., Chen S., Gao Q. (2019). Development of EST‐SSR markers in Saxifraga sinomontana (saxifragaceae) and cross‐amplification in three related species. Appl. Plant Sci..

[18] Chen Z., Liu Y.M., Yang S. (2008). Studies on the chemical constituents and anticancer activity of *Saxifraga stolonifera* (L) meeb. Bioorg. Med. Chem..

[19] T N., N W. (2016). Anti-cancer effect of Saxifraga stolonifera meerb. Clin. Exp. Pharmacol..

[20] Ohta S., Aoki T., Hirata T., Suga T. (1984). The structures of four diarylheptanoid glycosides from the female flowers of Alnus serrulatoides. J. Chem. Soc, Perkin Trans..

[21] Takesono A., Heasman S.J., Wojciak-Stothard B., Garg R., Ridley A.J. (2010). Microtubules regulate migratory polarity through Rho/ROCK signaling in T cells. PLoS One.

[22] Wang J., He Z., Wang G. (2022). Efficient targeted insertion of large DNA fragments without DNA donors. Nat. Methods.

[23] Kano Y., Gebregiworgis T., Marshall C.B. (2019). Tyrosyl phosphorylation of KRAS stalls GTPase cycle via alteration of switch I and II conformation. Nat. Commun..

[24] Claff T., Ebenhoch R., Kley J.T., Magarkar A., Nar H., Weichert D. (2025). Structural basis for lipid-mediated activation of G protein-coupled receptor GPR55. Nat. Commun..

[25] Ma Q., Surya W., He D. (2024). Spa2 remodels ADP-Actin via molecular condensation under glucose starvation. Nat. Commun..

[26] Dupont S., Morsut L., Aragona M. (2011). Role of YAP/TAZ in mechanotransduction. Nature.

[27] Kumar A., Wei Q., Yang R. (2024). *RHOAG17V* mutation exhibits markedly different functions in the presence of *TET2* loss in T-Cells. Blood.

[28] Zhang D., Liu L., Wang J. (2022). Drug-loaded PEG-PLGA nanoparticles for cancer treatment. Front. Pharmacol..

