# Supplementary material for: Mechanism of SC targeting RhoA regulation and its potential value in gastric cancer therapy
Source: Biochem Biophys Rep. 2025 Jul 24;43:102158. doi: 10.1016/j.bbrep.2025.102158 (PMC12311952; doi:10.1016/j.bbrep.2025.102158)

E-cadherin(proteintech, 20874-1-AP,1:5000, 125kDa)

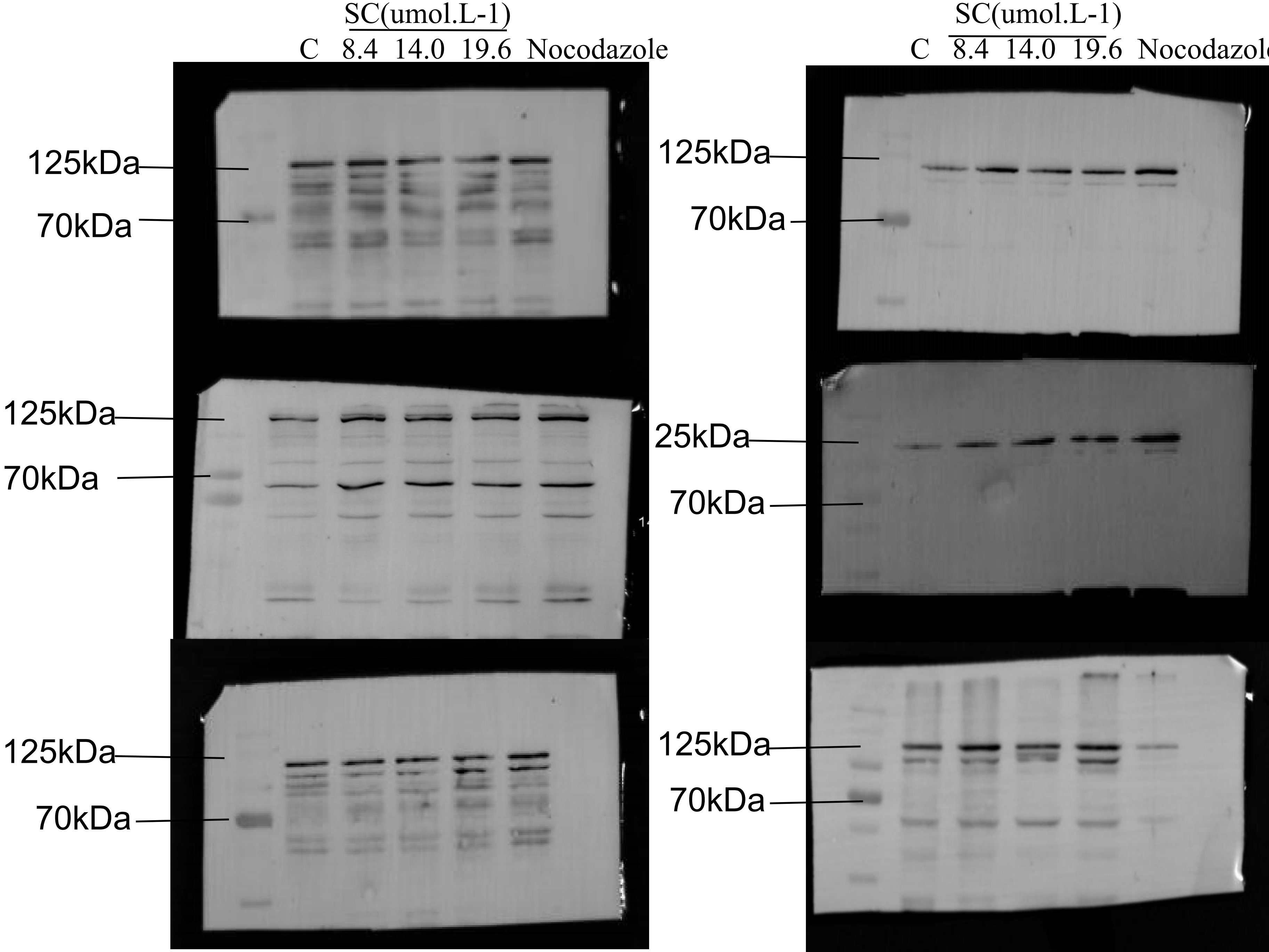

N-cadherin(proteintech, 66219-1-lg,1:5000, 130kDa)

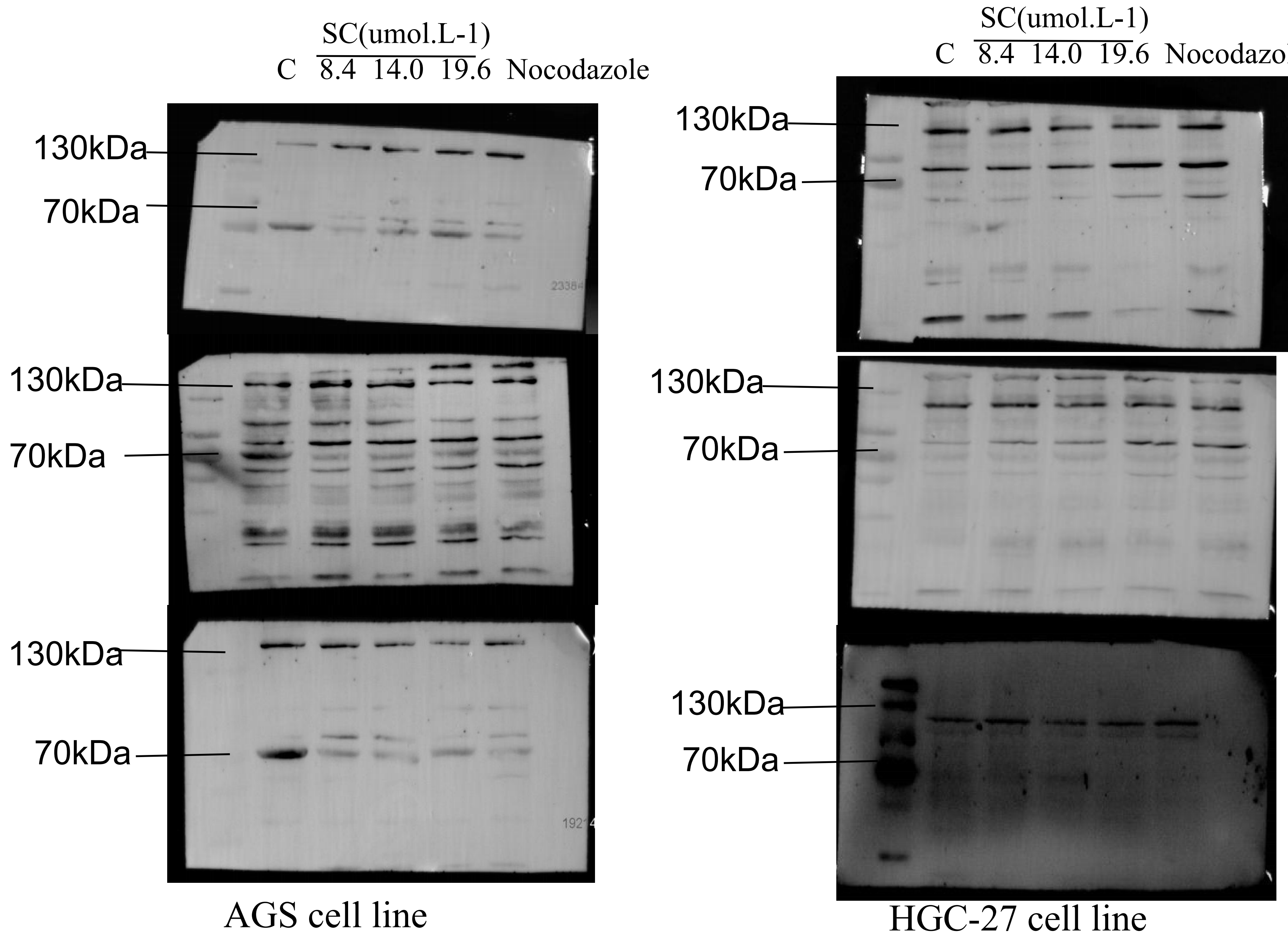

Vimentin(proteintech, 10366-1-AP,1:2000, 54kDa)

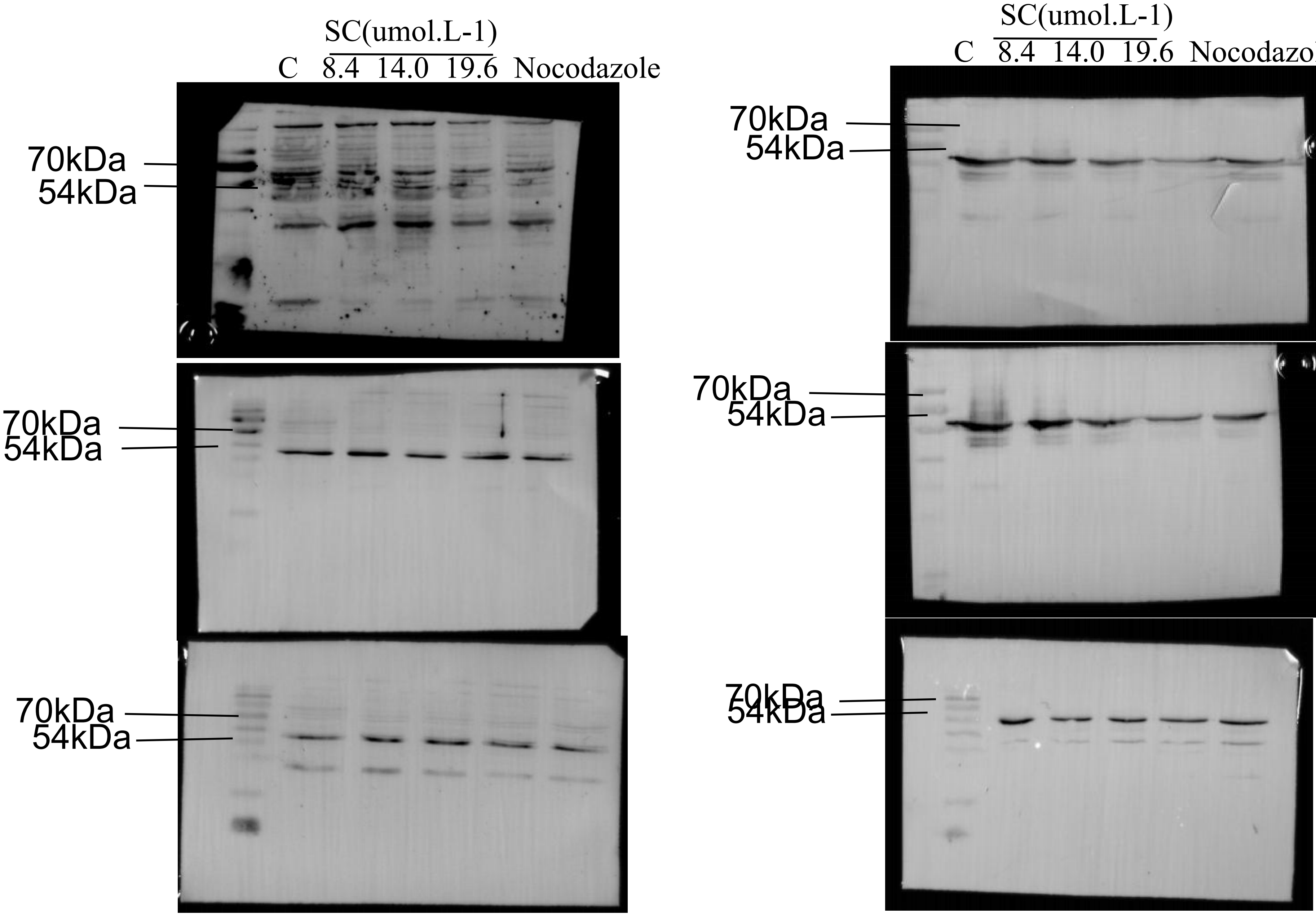

AGS cell line

HGC-27 cell line

Vimentin(proteintech, 10366-1-AP,1:2000, 54kDa)

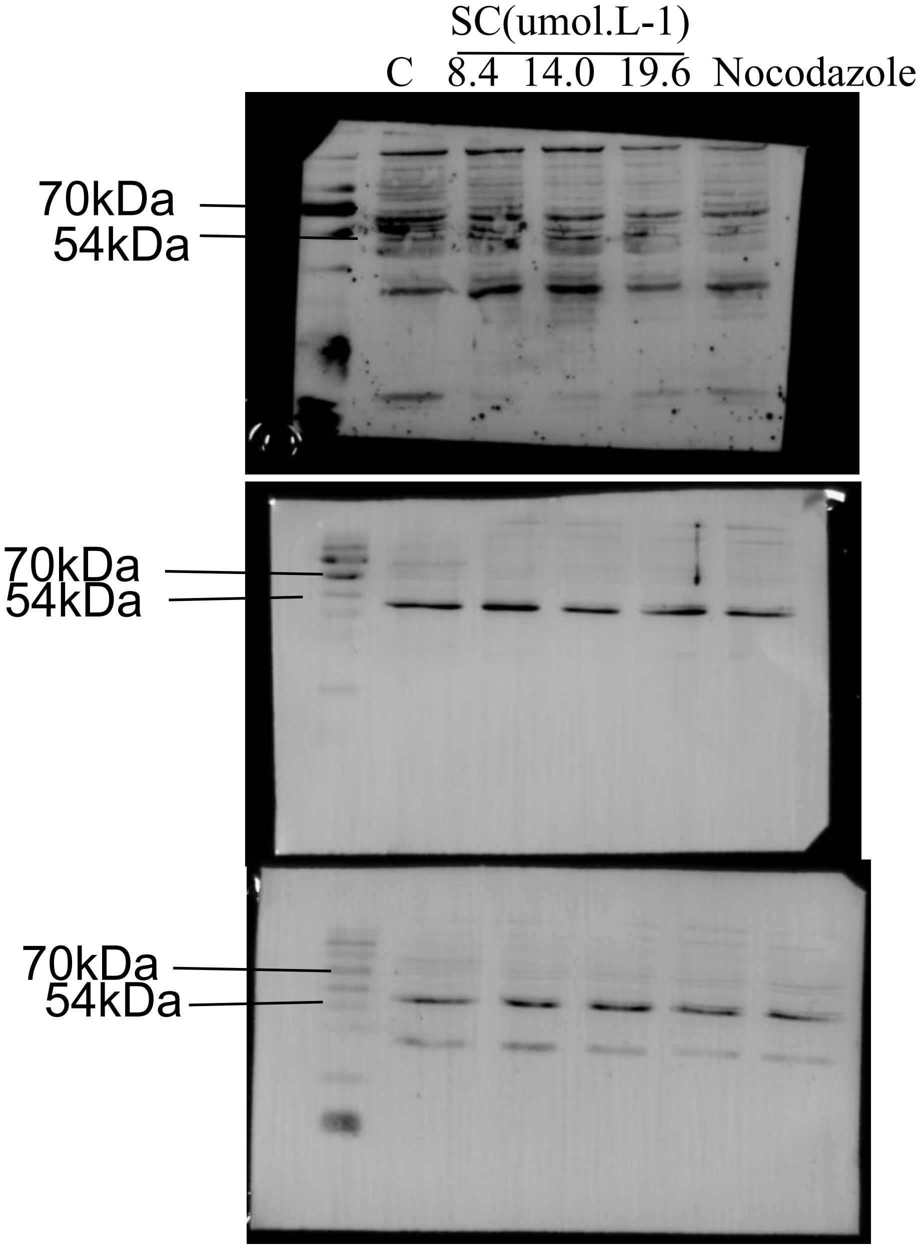

AGS cell line

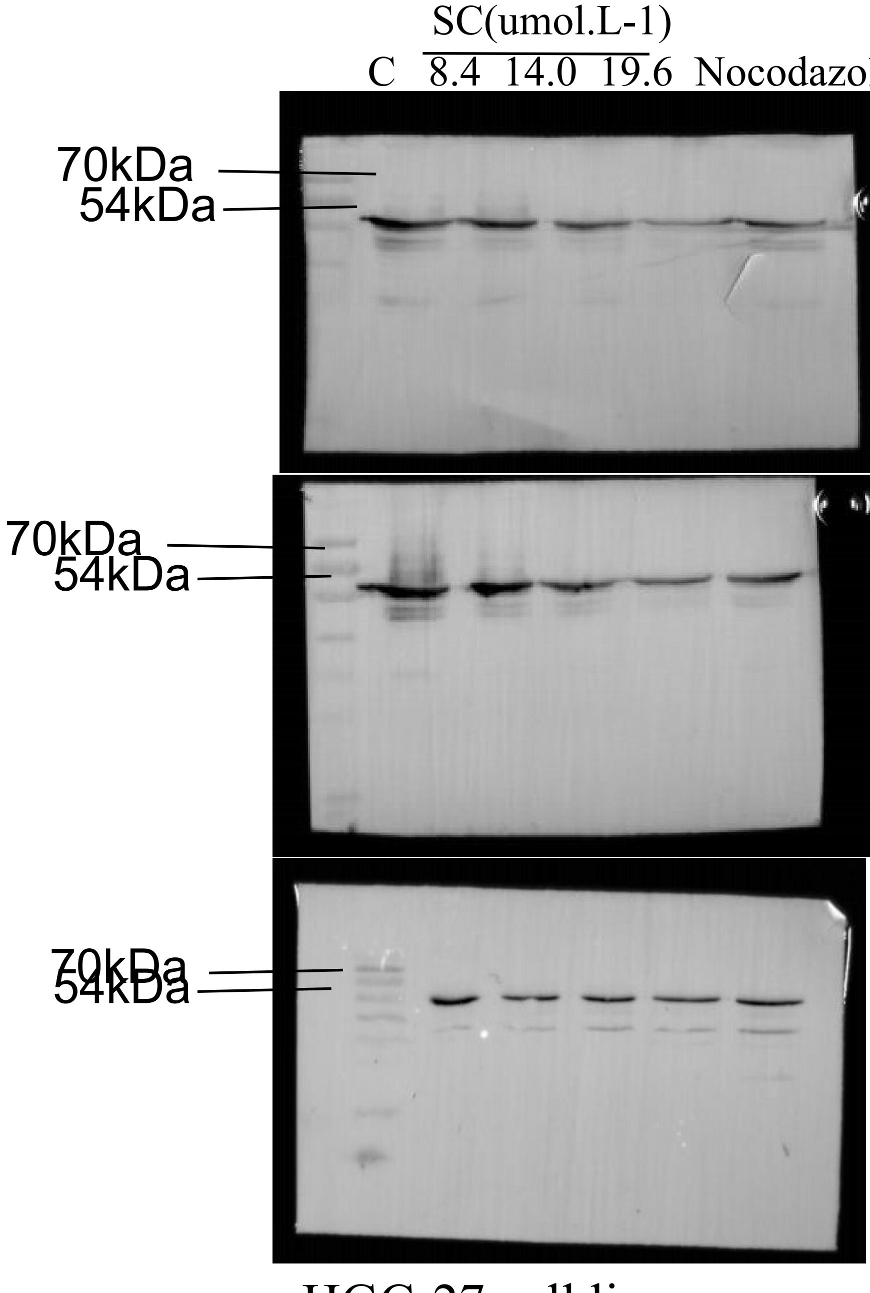

HGC-27 cell line

RhoA(proteintech, 10749-1-AP,1:500, 22kDa)

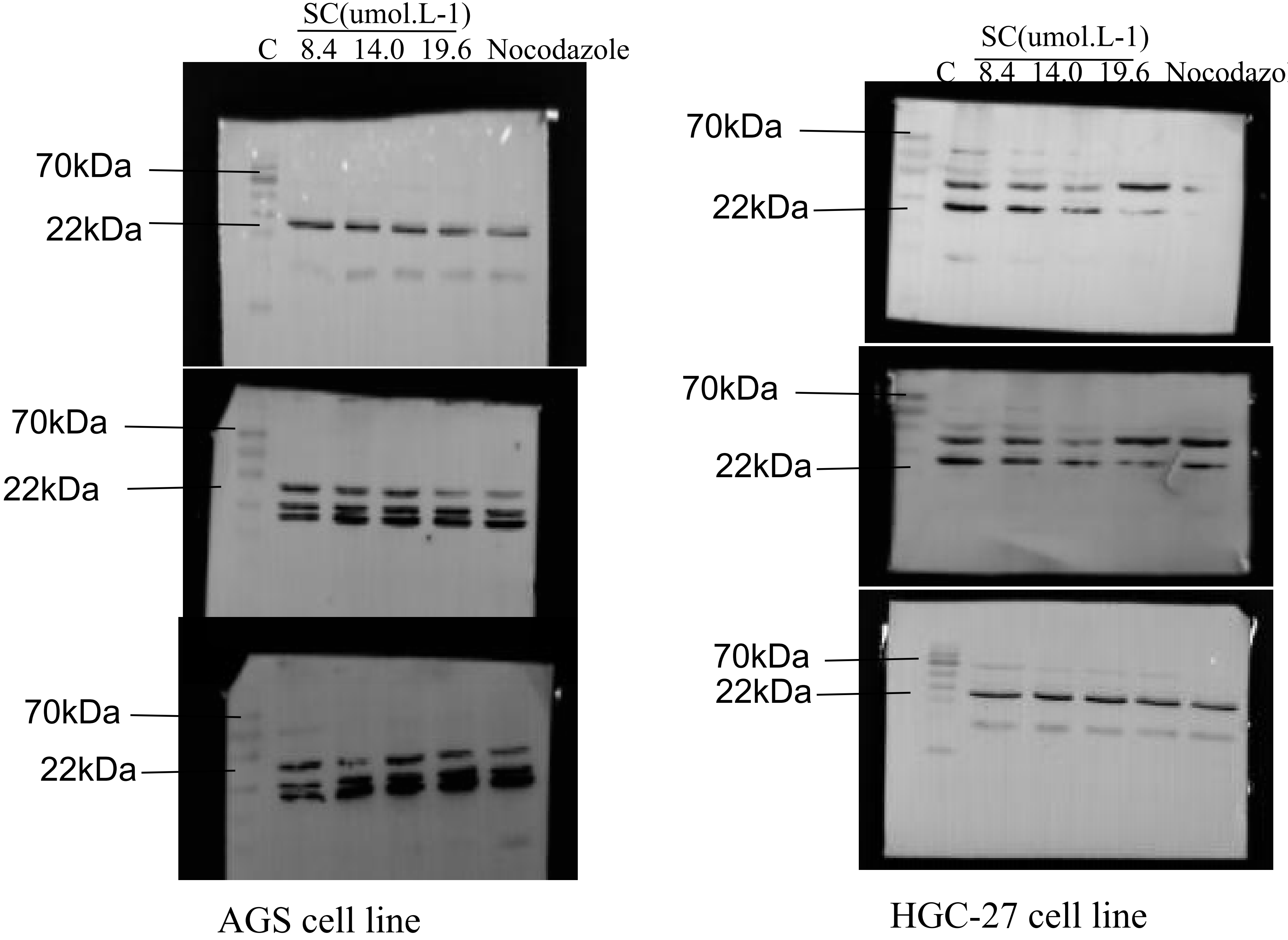

β-actin (proteintech, 66009-1-1g, 1:20000，42kDa)

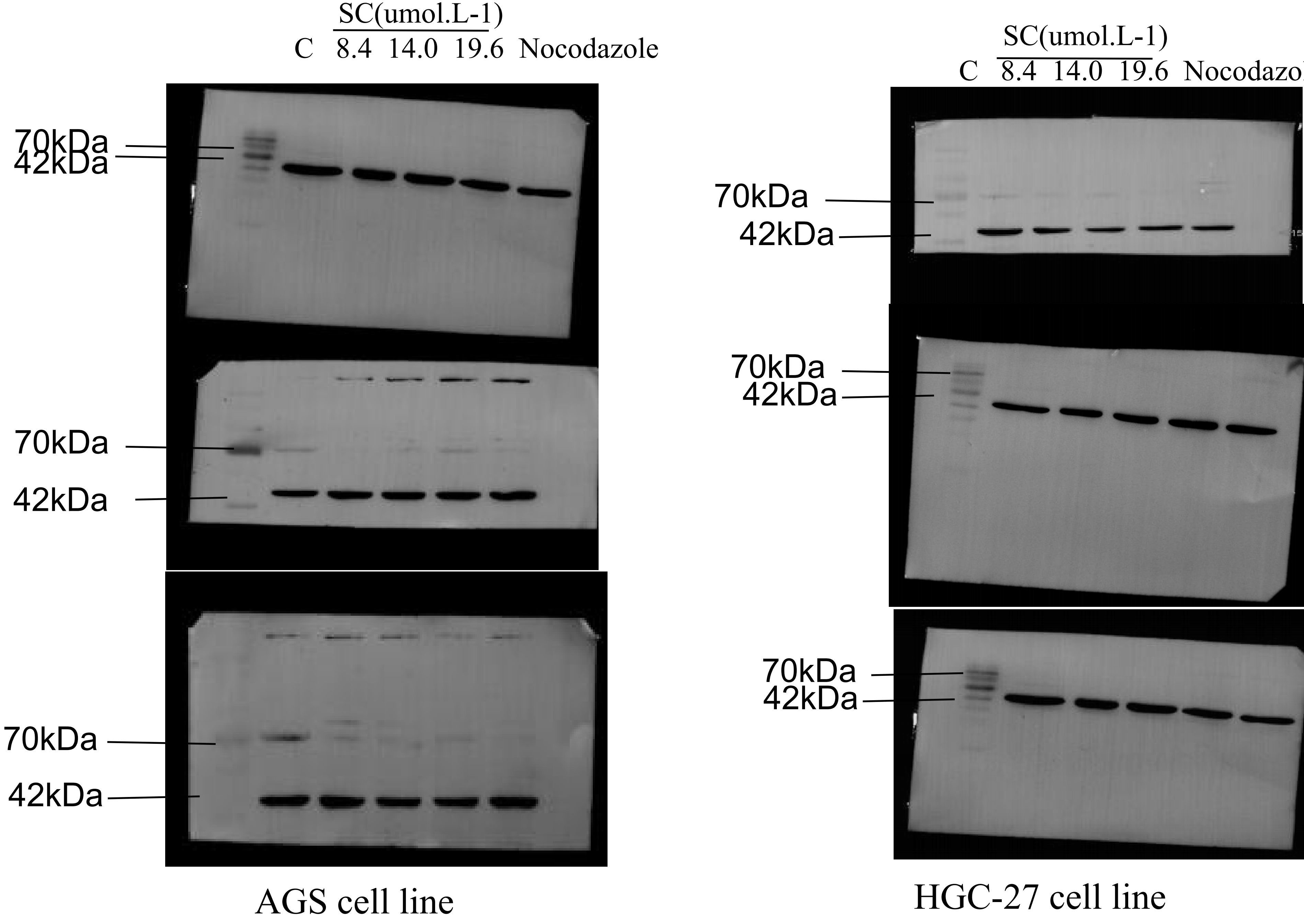

E-cadherin(proteintech, 20874-1-AP,1:5000, 125kDa)_Animal

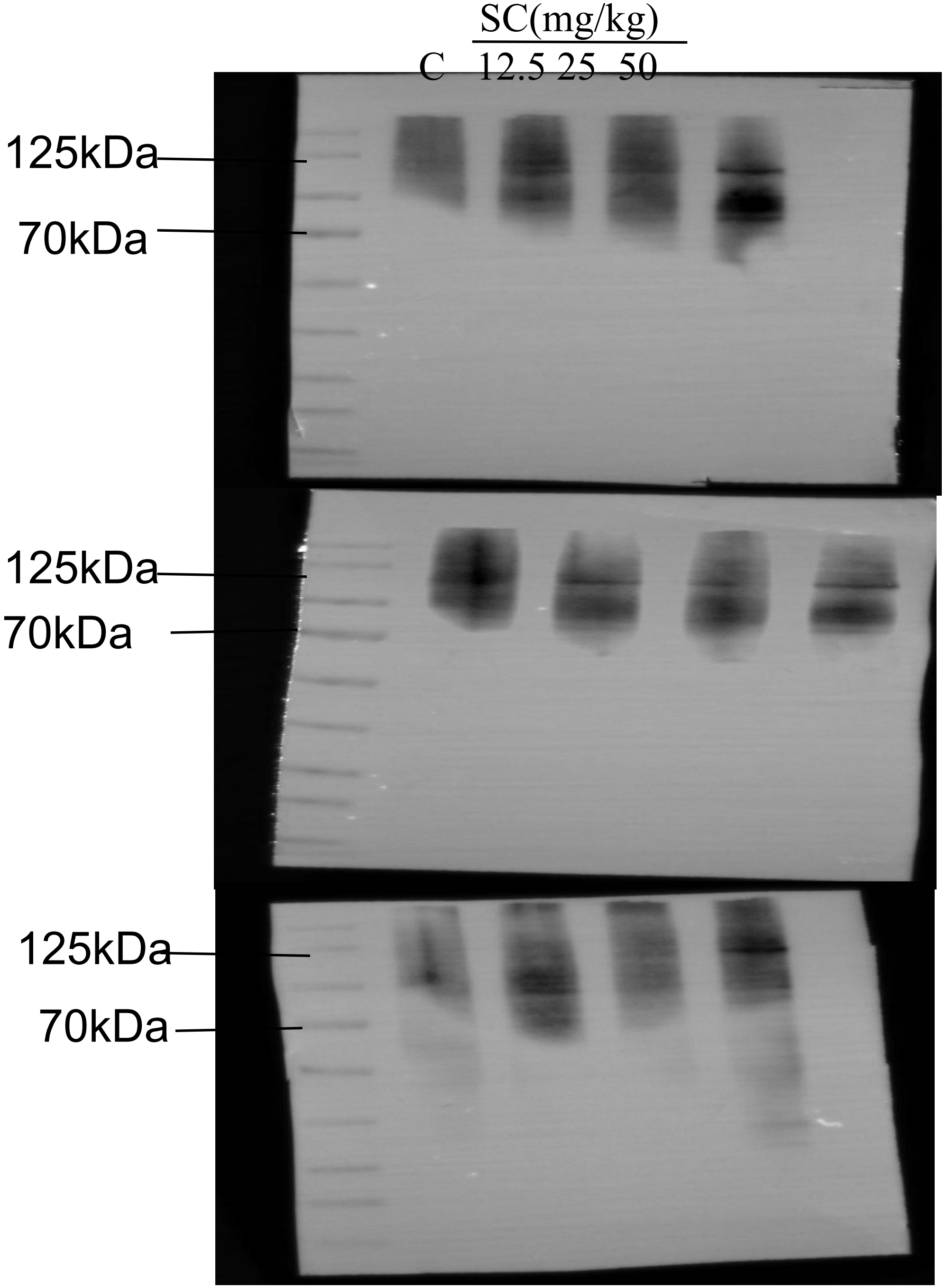

Vimentin(proteintech, 10366-1-AP,1:2000, 54kDa)_Animal

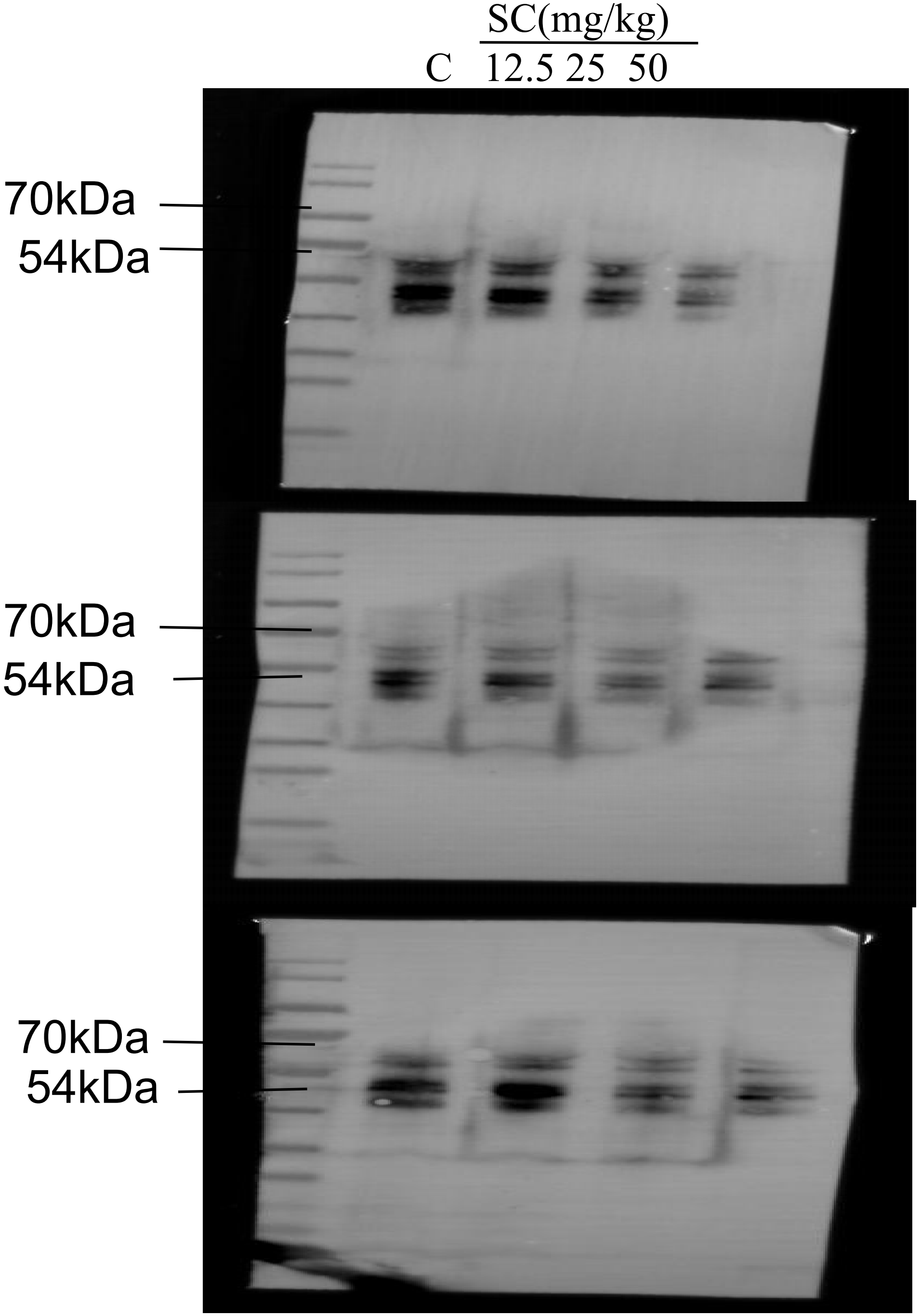

RhoA(proteintech, 10749-1-AP,1:500, 22kDa)_Animal

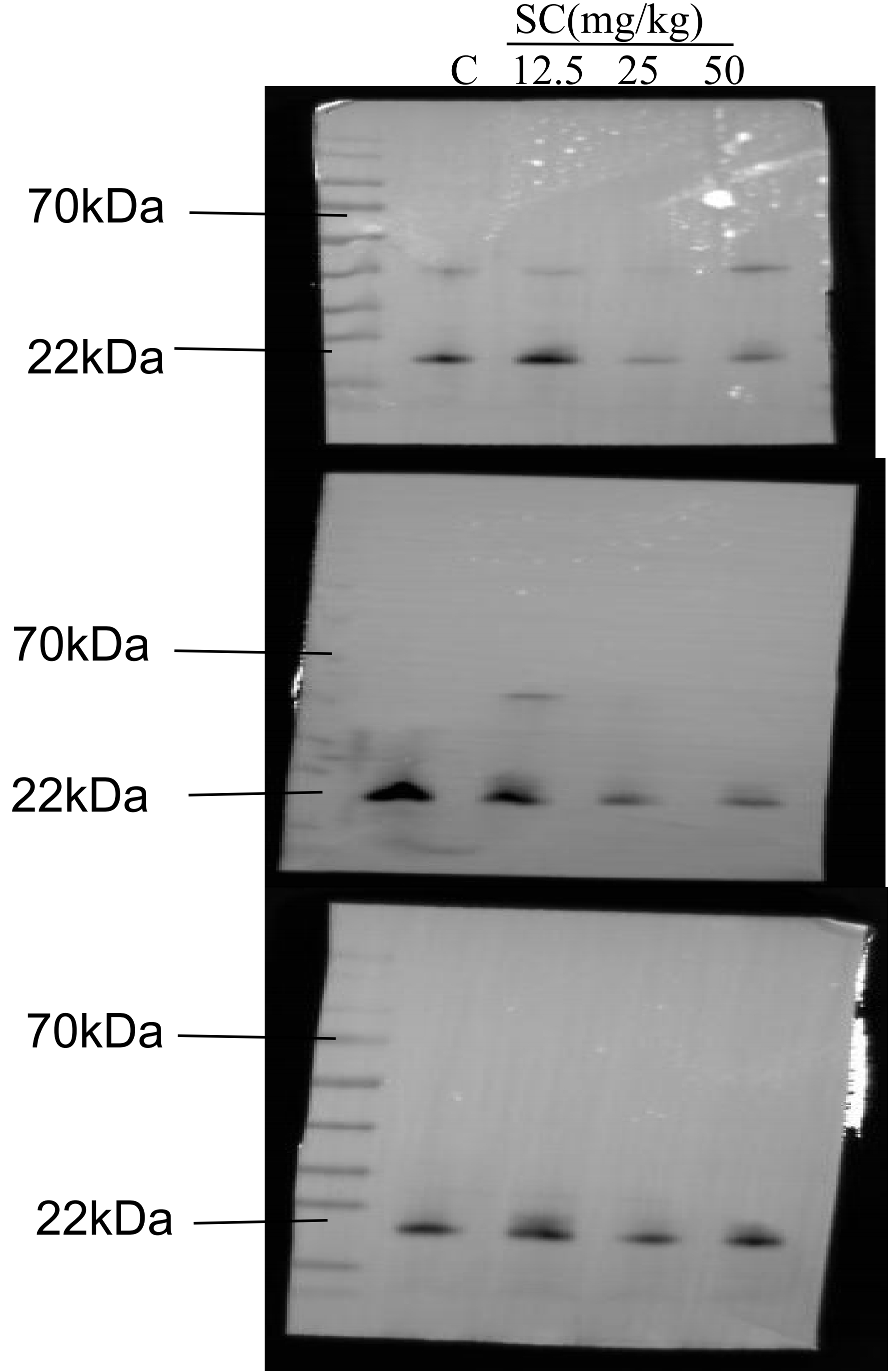

β-actin (proteintech, 66009-1-1g, 1:20000，42kDa)_Animal

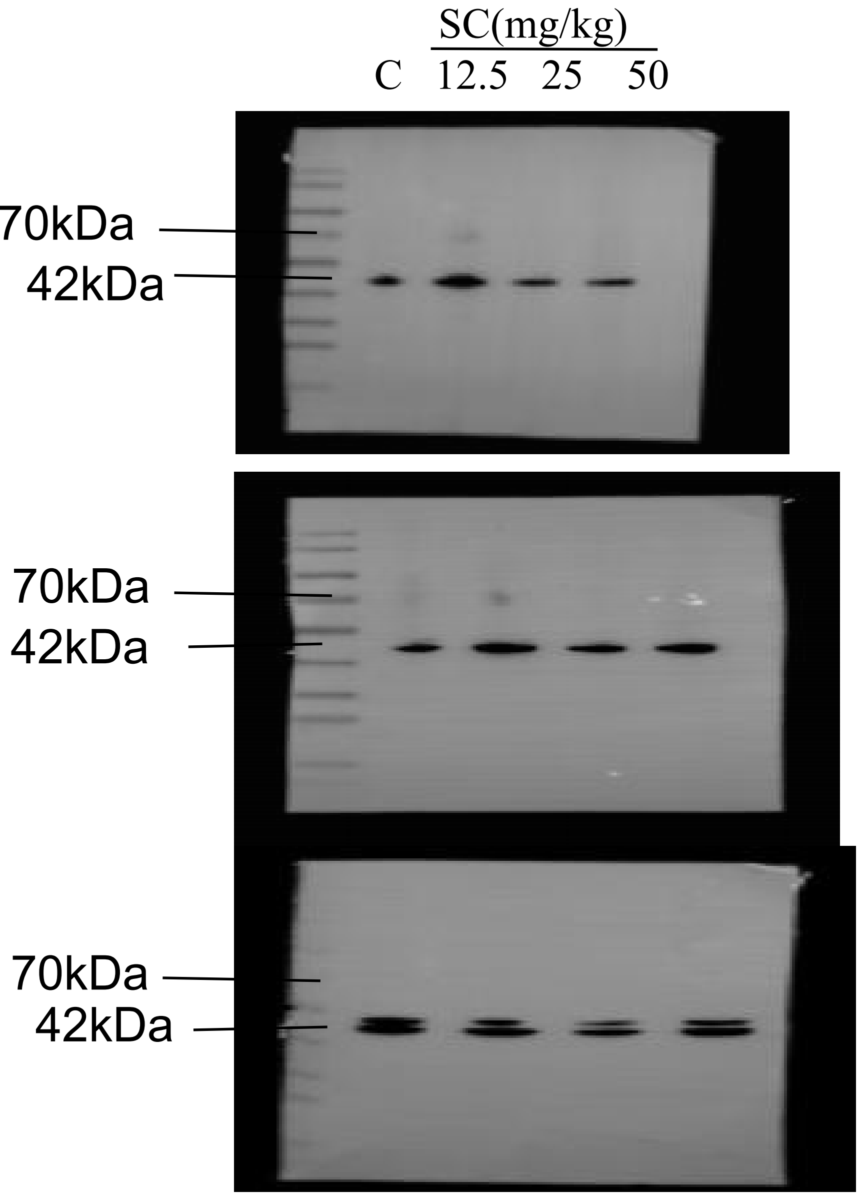

Supplement: Multimedia component 1 [file mmc1.pdf]
